# Evaluation of fecal immunoassays for canine *Echinococcus* infection in China

**DOI:** 10.1371/journal.pntd.0008690

**Published:** 2021-03-15

**Authors:** Liying Wang, Qian Wang, Huixia Cai, Hu Wang, Yan Huang, Yu Feng, Xuefei Bai, Min Qin, Sylvie Manguin, Laurent Gavotte, Weiping Wu, Roger Frutos

**Affiliations:** 1 National Institute of Parasitic Diseases, Chinese Center for Disease Control and Prevention; Chinese Center for Tropical Diseases Research; WHO Collaborating Centre for Tropical Diseases; National Center for International Research on Tropical Diseases, Ministry of Science and Technology; Key Laboratory of Parasite and Vector Biology, Ministry of Health, Shanghai, China; 2 HydroSciences Montpellier (HSM), Institut de Recherche pour le Développement (IRD), CNRS, Université Montpellier, Montpellier, France; 3 ISEM, UMR 5557, Université de Montpellier, Montpellier, France; 4 Cirad, UMR 17, Intertryp, Campus international de Baillarguet, Montpellier Cedex 5, France; 5 IES, Université Montpellier, CNRS, Montpellier Cedex 5, France; 6 Sichuan Provincial Center for Disease Control and Prevention, Chengdu, China; 7 Qinghai Provincial Institute for Endemic Disease Prevention and Control, Xining, China; 8 Gansu Provincial Center for Disease Control and Prevention, Lanzhou, China; IRCCS Sacro Cuore Don Calabria Hospital, ITALY

## Abstract

Human echinococcosis is present worldwide but it is in China that disease prevalence is the highest. In western China, especially in the Tibetan Plateau, the burden of echinococcosis is the most important. Dogs are a major definitive host of *Echinococcus* and monitoring the presence of *Echinococcus* worms in dogs is therefore essential to efficiently control the disease. Detection kits based on three different technologies including sandwich ELISA, (indirect) ELISA, and gold immunodiffusion, are currently marketed and used in China. The objective of this work was to assess the efficacy of these kits, in particular with respect to sensitivity and specificity. Four fecal antigen detection kits for canine infection reflecting the three technologies were obtained from companies and tested in parallel on 220 fecal samples. The results indicate that the performance is lower than expected, in particular in terms of sensitivity. The best results were obtained with the sandwich ELISA technology. The gold immunofiltration yielded the poorest results. In all cases, further development is needed to improve the performance of these kits which are key components for the control of echinococcosis.

## Author summary

Although present worldwide, human echinococcosis is at its highest prevalence in western China and particularly in the Tibetan Plateau. Controlling echinococcosis is a national priority and routine monitoring must be established. Dogs are the main infection source in China and surveying *Echinococcus* worms in dogs is therefore a research priority. Commercial detection kits are currently in use in China to monitor the presence of *Echinococcus* in dogs. The kits are based on three different technologies including sandwich ELISA with two monoclonal antibodies, (indirect) ELISA, and gold immunodiffusion. National survey programmes are essential for the control of echinococcosis and it is thus very important to assess the efficacy of these kits for the programmes. This work was therefore undertaken to assess efficacy of the kits, in particular with respect to sensitivity and specificity. Four fecal antigen detection kits for canine infection reflecting the three technologies were obtained from companies and tested in parallel on 220 fecal samples. The performance was lower than expected, in particular for their sensitivity which ranged between 51.5% and 83.9% with only two samples displaying a worm burden lower than 100. Three out of four kits showed non-specific cross-reactions with other parasites. The best results were obtained with the sandwich ELISA technology, whereas gold immunofiltration yielded the poorest results. However, in all cases, further development is strongly needed to improve the performance of these kits which are key components for the control of echinococcosis.

## Introduction

Echinococcosis is a health-threatening parasitic zoonotic disease caused by the larval stage of *Echinococcus* tapeworms [1]. Cystic echinococcosis (CE) and alveolar echinococcosis (AE) in humans, livestock, and small mammals, are triggered by the involuntary consumption of *Echinococcus granulosus* and *Echinococcus multilocularis* eggs, respectively, excreted in the feces of definitive hosts (e.g., carnivores). Naturally, the transmission occurs between definitive hosts (primarily dogs and foxes) and intermediate hosts (livestock and small mammals), whilst humans are accidental hosts. Human infection can occur through direct contact with definitive hosts or indirectly through contamination of food or possibly water with parasite eggs [2]. Echinococcosis has been recognized as one of the world’s public health issues. In humans, metacestode infection causes severe disease and possibly death. It also results in economic losses from treatment costs, lost wages, and livestock-associated production losses.

Both CE and AE are endemic in the pasture areas of western China, threatening more than 50 million people with a global echinococcosis prevalence of 0.28% in humans, 4.68% in livestock, and 4.25% in dogs. The number of patients was estimated to be 166,098 in 2016 [3]. Echinococcosis has been listed as a key parasitic disease in China [4,5]. Dog management and monthly deworming with praziquantel are two major intervention measures implemented to prevent human and livestock infections.

Infection rate in dogs is an important indicator to assess control efficacy and risk of disease transmission [6]. Coproantigen ELISA, often combined with mass ultrasound screening programmes for human CE, has been the preferred approach for monitoring and surveillance in resource-poor endemic areas and during control schemes [7]. Dogs infection rate is also a sensitive indicator to assess the risk and burden of echinococcosis and to evaluate the impact of control measures [8].

Currently, two sandwich ELISA kits and one indirect ELISA test (hereinafter referred to as ELISA) for the detection of *Echinococcus* coproantigen [9], as well as a gold immunofiltration assay, are commercially available in China. In this work, we evaluated the relative performance of these four kits which represent three different technologies in the detection of *Echinococcus granulosus* infections in dogs, in order to provide a reference for practical implementation in control programmes.

## Materials and methods

### Ethics statements

The 34 true positive samples collected by necropsy of infected dogs were approved by the ethics committee of the following institutions: Xinjiang Academy of Animal Sciences (24 samples), the First Affiliated Hospital of Xinjiang Medical University (six samples), and the Qinghai Provincial Institute for Endemic Disease Prevention and Control (four samples). The animal trial was approved following institutional ethical guidelines by the ethics committee at the First Affiliated Hospital of Xinjiang Medical University and has followed the code by the ethics committee of ZSSOM on Laboratory Animal Care.

### Detection kits

This study assessed four kits that are currently being used for the prevention and control of echinococcosis in China. The kits were randomly coded as A, B, C, and D. The information on the kits is provided in Table 1. These kits were provided by Xinjiang Tecon Animal Husbandry Bio-Technology Co. Ltd (kit A), Zhuhai S.E.Z. Haitai Biological Pharmaceuticals Co. Ltd (kits B and C), and Shenzhen Combined Biotech Co. Ltd (kit D) (Table 1). Two kits were sandwich ELISA tests (A and B), one is an ELISA test (D), and one is a gold immunofiltration assay (C) (Table 1).

**Table 1:** Major features of the assessed tests for *Echinococcus granulosus* diagnosis in dogs.

Code	Type	Sample volume	Extra supplies	Time required	Manufacturer
A	Sandwich ELISA 2 MAbs	100 μL	No	135 min	Xinjiang Tecon Animal Husbandry Biotechnology Co. Ltd
B	Sandwich ELISA 2 MAbs	100 μL	No	135 min	Zhuhai S.E.Z. Haitai Biological Pharmaceuticals Co. Ltd
C	Gold immunofiltration	300 μL	No	130 min	Zhuhai S.E.Z. Haitai Biological Pharmaceuticals Co. Ltd
D	ELISA	100 μL	No	150 min	Shenzhen Combined Biotech Co. Ltd

### Collection of specimens

A total of 34 positive canine fecal specimens were collected from dogs in Xinjiang and Qinghai. Positive cases were identified by demonstration for the presence of adult worms in the intestine, which is considered as the “gold” standard for the identification of *Echinococcus* infections [10]. Hence, we detected *E*. *granulosus* from 34 dogs that were then euthanized and, infection was confirmed and parasite burden recorded ranged between 5 and 25,000 worms (Table 2). A complement of 158 negative canine fecal specimens were collected, out of which 116 were from non-endemic areas in Gansu and 42 from laboratory dogs without any parasitic infection. An additional 28 samples of canine fecal specimens were also collected from dogs displaying other parasitic infections. Eight samples of *Taenia hydatigera*, eight of *Dipylidium caninum*, and 12 of *Spirometra mansoni*, were collected in the Guangdong Province (Table 3). All specimens were verified by etiologic inspection.

**Table 2:** Specific parasite burden in the positive samples (n = 34).

Infection Level	Worm burden range	Number of infected dogs	Worm burden
I	[0–500]	9	5; 60; 100; 200(3); 300(2); 400
II	[500–5,000]	10	600(2); 1,100; 1,500; 2,100; 3,100 (2); 3,500(2); 4,000
III	[5,000–20,000]	7	6,000(5); 11,000(2)
IV	[20,000-+∞]	8	20000(3); 25000(5)

**Table 3:** Composition and origin of samples.

Category	Number of dogs sampled	Sample origin
Positive canine fecal specimens	34 (*Echinococcus granulosus* infection)	Qinghai Provincial Institute for Endemic Disease Prevention and Control The Animal Husbandry Institute of Xinjiang Uygur Autonomous Region
Negative canine fecal specimens	116 from non-endemic areas 42 from laboratory dogs	Gansu Center for Disease Control and Prevention First Affiliated Hospital of Xinjiang Medical University.
Canine fecal specimens of other parasitic infections	28 (including eight with *Taenia hydatigena*, eight with *Dipylidium caninum*, and 12 with *Spirometra mansoni*)	Sun Yat-Sen University of Guangdong Province

### Preparation of samples

A double-blind method was used in the detection process. Experimenters did not know what they were testing; they only received code numbers as sample identifiers. In order to ensure that the concentration of sample in the different groups was the same, the preliminary preparation of the samples was performed by a senior experimenter. Samples were stored at -80°C upon collection. Fecal specimens were defrosted and 3 g of each sample were diluted in phosphate buffer saline (PBS) at pH 7.2~7.4, to a final concentration of 1 g/mL and centrifuged at 3,000 g for 30 minutes. After centrifugation, 2 mL of supernatant were collected. For two groups of parallel samples for each kit, six sample batches of 100 μL and two sample batches of 300 μL were prepared. In order to avoid any mutual confirmation of results, all samples were randomly encoded for each group. So, there were different coding orders for eight groups of samples.

### Detection tests

All samples were tested with each kit in duplicate (to enable statistical analysis of data), according to the manufacturer’s instructions. In order to reflect accuracy of the kits, each detection test was performed by an operator assigned by the company for each kit. Parallel detection tests with the four different kits were conducted simultaneously in the same laboratory.

### Analysis of data

Data were analyzed using the SPSS 20.0 software package (IBM, Armonk, USA). The indicators considered for analysis were: accuracy, reliability, sensitivity, specificity, positive predictive value, negative predictive value, Youden’s index, cross reaction rate, consistency rate, Kappa value, and Repeatability. We randomly selected two parallel groups including 34 fecal specimens and calculated the average as the sensitivity of each detection method. 95% CI were calculated by Clopper-Pearson approach according to sample size. Significance of the data were tested using chi-square test. Each index is the average of the test results of two groups of parallel samples. Definitions and calculation methods of relevant indicators are as follows.

#### Sensitivity

Proportion of known infected fecal samples that tested positive in an assay (infected fecal samples that tested negative are considered as false negatives).

#### Specificity

Proportion of uninfected reference fecal samples that tested negative in an assay (uninfected fecal samples that tested positive are regarded as false positives). This type of specificity is denominated **specificity 1**. Specificity tests which referring to reference fecal samples not infected with *Echinococcus* but harboring other parasites is denominated **specificity 2.** The specificity we calculated in this study belongs to type specificity 1.

#### Cross reaction rate

Proportion of samples uninfected with *Echinococcus* but harboring other parasites reference fecal samples, which tested positive in an assay.

#### Positive predictive value (PV+)

PV+ is an indicator of the probability that individuals with positive testing results do have the disease.

#### Negative predictive value (PV-)

The PV- is an indicator of the probability that individuals with negative testing results do not have the disease.

#### Youden’s index

Youden’s index expresses the total ability of a reagent to detect true positive or true negative samples.

#### Consistency rate

Proportion of samples with the same test results of reagents as the real results.

#### Kappa value

Kappa value was used to analyze and evaluate the consistency of two parallel samples detected by one detection method, considering the influence of opportunity factors on consistency rate.

Sensitivity = $\frac{TP}{TP+FN}$×100%

Specificity = $\frac{TN}{FP+TN}$×100%

PV+ = $\frac{TP}{TP+FP}$×100%

PV- = $\frac{TN}{TN+FN}$×100%

Youden’s index = (Sensitivity+Specificity)-1

Cross reaction rate = 1- specificity 2

Consistency rate = $\frac{TP+TN}{N}$×100%

Kappa value = $\frac{N(\mathrm{TP}+\mathrm{TN})-(R1C1+R2C2)}{N^{2}-(R1C1+R2C2)}$×100%

#### Repeatability

For a test reagent, the percentage of samples with consistent results in two groups of parallel samples.

N: total number of samples; TP: true positive; FP: false positive; TN: true negative; FN: false negative; R1: sum of the first row; R2: sum of the second row;

C1: sum of the first column; C1: sum of the second column.

Sensibility and specificity data were circularized (arc sinus transformation) and normality was confirmed by Kolmogorov Smirnov normality test.

## Results

### Sensitivity assessment

The sensitivity of each detection kit was assessed using the 34 feces obtained from *Echinococcus*-infected dogs listed in Table 4. Kit B displayed the highest average sensitivity, i.e., 83.82%; while D showed the lowest average sensitivity, i.e., 51.47% (Table 4, Table 5). The average sensitivity of kits A and C was 76.47% and 70.59%, respectively. When the sensitivity was calculated according to the worm burden, strong variations were observed (Table 5). The sensitivity varied widely depending on the worm count. For kit A, the sensibility varied from a lowest rate of 44.44% for a worm burden class of 500 or less to a maximum of 100% for a worm burden of 5,000 to 20,000. The sensitivity decreased sharply to 81% for a worm burden above 20,000 (Table 5). For the other three kits, the calculated sensitivity increased along with the worm burden. The lowest sensitivity for a worm burden below 500 was 72.22%, 44.44%, and 11.11% for kits, B, C, and D, respectively (Table 5). The highest sensitivity was observed for a worm burden above 20,000 with 93.75% for kits B and C, and 81.25% for kit A and D (Table 5).

**Table 4:** Summary of evaluation results for relevant indicators.

Assay code	Randomized ID	Sensitivity [95%CI]		Specificity [95%CI]		Positive predictive value [95%CI]		Negative predictive value [95%CI]		Youden’s index		Consistency rate		Kappa value	Repeatability
A	1	82.40% [65.50%, 93.20%]	76.47% [64.60%, 85.90%]	89.20% [83.30%, 93.60%]	87.97%	62.22% [46.50%, 76.20%]	57.78% [46.90%, 68.10%]	95.92% [91.30% ,98.50%]	94.56% [91.30%, 96.90%]	0.72	0.64	88.02%	85.94%	0.69	88.18%(194/220)
		(28/34)		(141/158)	-12.03%	(28/45)		(141/147)							
	5	70.60% [52.50%, 84.90%]		86.70% [80.40%, 91.60%]	[83.90%, 91.30%]	53.33% [37.90%, 68.30%]		93.20% [87.80% ,96.70]		0.57		83.85%			
		(24/34)		(137/158)		(24/45)		(137/147)							
B	3	82.40% [65.50%, 93.20%]	83.82% [72.90%, 91.60%]	72.80% [65.10%, 79.60%]	74.68%	39.44% [28.00%, 51.70%]	41.69% [33.30%, 50.30%]	95.04% [89.50% ,98.20%]	95.54% [92.20%, 97.80%]	0.55	0.59	74.48%	76.30%	0.65	84.55%(186/220)
		(28/34)		(115/158)	-25.32%	(28/71)		(115/121)							
	4	85.30% [68.90%, 95.00%]		76.60% [69.20%, 82.90%]	[69.50%, 79.40%]	43.94% [31.70%, 56.70%]		96.03% [91.00% ,98.70%]		0.62		78.13%			
		(29/34)		(121/158)		(29/66)		(121/126)							
C	7	67.60% [49.50%, 82.60%]	70.59% [58.30%, 81.00%]	63.30% [55.30%, 70.80%]	63.61%	28.40% [18.90%, 39.50%]	29.44% [22.60%, 37.10%]	90.09% [83.00%, 94.90%]	90.95% [86.40%, 94.40%]	0.31	0.34	64.06%	64.84%	0.63	82.73%(182/220)
		(23/34)		(100/158)	-36.39%	(23/81)		(100/111)							
	8	73.50% [55.60%, 87.10%]		63.90% [55.90%, 71.40%]	[58.00%, 68.90%]	30.49% [20.80%, 41.60%]		91.82% [85.00%, 91.20%]		0.37		65.63%			
		(25/34)		(101/158)		(25/82)		(101/110)							
D	2	52.90% [35.10%, 70.20%]	51.47% [39.00%, 63.80%]	74.70% [67.20%, 81.30%]	75.63%	31.03% [19.50%, 44.50%]	31.26% [22.80%, 40.70%]	88.06% [81.30%, 93.00%]	87.87% [83.40%, 91.50%]	0.28	0.27	70.83%	71.35%	0.64	87.73%(193/220)
		(18/34)		(118/158)	-24.37%	(18/58)		(118/134)							
	6	50.00% [32.40%, 67.60%]		76.60% [69.20%, 82.90%]	[70.50%, 80.30%]	31.48% [19.50%, 45.60%]		87.68% [81.00%, 92.70%]		0.27		71.88%			
		(17/34)		(121/158)		(17/54)		(121/138)							
Result			*X*^*2*^ = 9.31		*X*^*2*^ = 24.90		*X*^*2*^ = 40.20		*X*^*2*^ = 9.46				*X*^*2*^ = 23.44		

p value			*P*<0.05		*P*<0.05		*P*<0.05		*P*<0.05				*P*<0.05		

**Table 5:** Effect of worm burden sensitivity.

Assay code	Randomized sample ID	Worm burden										Number of false negatives	Worm count for false negatives
		[0–500]		[500–5,000]		[5,000–20,000]		[20,000-+∞]		Total			
		n = 9	Sensitivity (%) [95%CI]	n = 10	Sensitivity (%) [95%CI]	n = 7	Sensitivity (%) [95%CI]	n = 8	Sensitivity (%) [95%CI]	N = 34	Average sensitivity (%)		
A	1	5 55.60 [21.20,86.30]	44.44 [21.50, 69.20]	9 90.00 [55.50,99.70]	85.00 [62.10, 96.80]	7 100.00 [59.00,100.00]	100.00 [59.00, 100.00]	7 87.50 [47.30,99.70]	81.25 [54.40, 96.00]	28	76.47	6	5; 60; 100; 400; 1,500; 20,000
	5	3 33.30 [7.50,70.10]		8 80.00 [44.40,97.50]		7 100.00 [59.00,100.00]		6 75.00 [34.90,96.80]		24		10	5; 60; 100; 200; 300; 400; 2,100; 3,100; 20,000; 25,000
B	3	6 66.70 [29.90,92.50]	72.22 [46.50, 90.30]	8 80.00 [44.40,97.50]	80.00 [44.40, 97.50]	6 85.70 [42.10,99.60]	92.86 [66.10, 99.80]	8 100.00 [63.10,100.00]	93.75 [69.80, 99.80]	28	83.82	6	5; 60; 200; 600(2); 11,000
	4	7 77.80 [40.00,97.20]		8 80.00 [44.40,97.50]		7 100.00 [59.00,100.00]		7 87.50 [47.30,99.70]		29		5	60; 200; 600(2); 20,000
C	7	3 33.30 [7.50,70.10]	44.44 [21.50, 69.20]	7 70.00 [34.80,93.30]	65.00 [40.80, 84.60]	6 85.70 [42.10,99.60]	85.71 [42.10, 99.60]	7 87.50 [47.30,99.70]	93.75 [69.80, 99.80]	23	70.59	11	5; 100; 200(3); 400; 600(2); 1,100; 6,000; 25,000
	8	5 55.60 [21.20,86.30]		6 60.00 [26.20,87.80]		6 85.70 [42.10,99.60]		8 100.00 [63.10,100.00]		25		9	5; 100; 200(2); 600(2); 1,100; 3,100; 6,000
D	2	1 11.10 [0.30, 48.20]	11.11 [0.30, 48.20]	5 50.00 [18.70,81.30]	50.00 [18.70, 81.30]	5 71.40 [29.00,96.30]	71.43 [29.00, 96. 30	7 87.50 [47.30,99.70]	81.25 [54.40, 96.00]	18	51.47	16	5; 60; 100; 200(3); 300; 400; 600(2); 1,500; 2,100; 3,100; 6,000(2); 20,000
	6	1 11.10 [0.30, 48.20]		5 50.00 [18.70,81.30]		5 71.40 [29.00,96.30]		6 75.00 [34.90,96.80]		17		17	5; 60; 100; 200(3); 300; 400; 600(2); 1,500; 2,100; 3,100; 6000(2); 20,000(2)

### Assessment of false positives (specificity 1)

The level of non-specific reactions was assessed for each detection kit on 158 feces obtained from *Echinococcus-*negative dogs (Table 4). We randomly selected two parallel groups from the 158 fecal specimens. The lowest level of non-specific reaction was shown by kit A (12.03%), while kit C displayed the highest level of non-specificity (36.39%). Kits B and D yielded intermediate values of 25.32% and 24.37%, respectively (Table 4).

### Assessment of cross-reactivity with other tapeworms (specificity 2)

Kit A displayed no cross-reactivity at all with any of the control parasites, i.e., *T*. *hydatigena*, *D*. *caninum*, or *S*. *mansoni* (Table 6). Kits B and C displayed the highest level of cross-reactivity estimated at 23.21%, whereas kit D showed an intermediate level of 16.07%. Kit C cross-reacted with all three heterologous worms. Kit B showed cross-reactivity with *D*. *caninum* and *S*. *mansoni*, while kit D cross-reacted with *T*. *hydatigena* and *D*. *caninum*.

**Table 6:** Cross-reactivity with other parasites.

Assay code	Randomized sample ID	Number of positive tests				Cross reaction rate (%)
		*Taenia hydatigena* n = 8	*Dipylidium caninum* n = 8	*Spirometra mansoni* n = 12	Total N = 28	
A	1	0	0	0	0	0.00
	5	0	0	0	0	
B	3	0	3	3	6	23.21
	4	0	4	3	7	
C	7	3	2	2	7	23.21
	8	6	0	0	6	
D	2	1	5	0	6	16.07
	6	1	2	0	3	

### Assessment of global performance and accuracy

The best score when using Youden’s index was obtained by kit A (0.64), whereas kit B reached a score of 0.59 (Table 4). Kits C and D obtained very low scores of 0.34 and 0.27, respectively (Table 4). The Youden’s index varies from 0 to 1, with 0 indicating an undiscriminating, therefore useless test, while 1 indicates a perfect test. Even with the best scores, kits A and B were far from being perfect. The accuracy assessment conducted to evaluate the repeatability of each test yielded scores higher than 80% whatever the kit considered. However, kits A and D reached higher scores of 88.18% and 87.73%, respectively, compared to kits B and C scores which were 84.55% and 82.73%, respectively.

## Discussion

Owing to their effectiveness, ELISA tests have been introduced to local echinococcosis prevention programmes where they are currently being implemented. ELISA has been adopted as the main diagnostic method in place of the arecoline cathartic method to monitor canine *Echinococcus* infection in control programmes. There is thus a need for regular evaluation of fecal antigen tests in order to improve the quality of monitoring activities and objectively assess prevention effectiveness.

In this work, we evaluated the accuracy and reliability of four commercial kits currently in use in China and results showed that the sensitivity of the four kits ranged between 51.5% and 83.9% only. Out of all the samples tested, only two displayed a worm burden lower than 100. Thus, the range of sensitivity obtained in this study is far below that from a previous study which reported between 83% and 100% for an average of more than 1,000 worms for fecal anti detection [11]. A sensitivity ranging between 29% and 79% has been previously reported for a worm burden lower than 100 worms as determined by necropsy or arecoline cathartic [10]. This is more in the range of what was observed in this work, but with a worm burden higher than 100. The sensitivity results found in this work also indicates that the threshold of 100 is not realistic, which could explain the variation in results from one report to another. Thus, we estimate that the minimal burden of worms for assessing sensitivity might be 500. Nevertheless, owing to the quite low sensitivity observed and the important variation induced by the worm burden, further modifications or optimization must be taken into consideration to increase the sensitivity of the kits we tested.

Non-specific or false positive reactions from the kits tested in this study ranged between 12.03% and 36.39%, while cross-reactivity with other parasites were from 0 to 23.21%, depending upon the kit. Youden’s index is a comprehensive indicator that reflects sensitivity and specificity. Under the assumption that sensitivity and specificity are equally important, the kit with the highest Youden’s index is given priority. In this study, the highest Youden’s index was 0.64 for kit A. However, the positive predictive value changes with the infection rate. In addition, the detection results corresponding to the infection rate of 17.70% (34/192) were generally low, indicating the occurrence of false positive results. The consistency rate is the main index reflecting the reliability of kits, which mainly represents the stability of the detection ability of kits. The highest consistency rate of kit A is 85.94%. Kappa value is also an important index that reflects the repeatability of test results. Thus, kit A showed the best reliability in terms of repeatability and stability; however, although quite high, there is a need for further improvement because the proportion of false positives is still high.

Out of the three technologies assessed, i.e., sandwich ELISA, ELISA, and immunofiltration, the latter displayed the lowest performance score. Immunofiltration has the advantage of being used *in situ* with a simple protocol and results being immediately available. However, the poor performance displayed by this technology does not make it a reliable and efficient choice for the monitoring of echinococcosis. More developments are therefore needed to improve this technology. Three of these tests, i.e., kits A, B, and D, have been previously assessed but with a smaller sample size [11]. Findings from the study had revealed that, the ELISA kit (kit D) yielded the sensibility but lacked specificity. Conversely, kit A displayed the best specificity but lacked sensitivity, while kit B gave intermediate results. In the current study, the results are totally different and clearly show that sandwich ELISA, i.e., kits A and B, are the appropriate methodology to implement for the surveillance of canine echinococcosis. Kit A displayed a better mean score than kit B. However, although lower, the latter yielded a very close score. It is thus difficult to discriminate kits A and B, since they are both based on the technology of sandwich ELISA with two monoclonal antibodies. ELISA, i.e., kit D, showed performances intermediate between sandwich ELISA and immunofiltration, and does not appear as a reliable option for surveillance. Nevertheless, even if sandwich ELISA seems to be the technology of choice for the surveillance of canine echinococcosis, improvements and optimization are still needed to ensure proper surveillance.

Several studies have been conducted in other countries but with different kits, procedures, and epitopes, making the comparison more difficult than between this study and that reported in 2014 [11]. Nevertheless, sandwich ELISA was found to be the most effective in Sardinia [12]. CoproELISA was compared to serum ELISA in Spain [13]. CoproELISA yielded a better sensitivity but included false positives [13]. CoproELISA was confirmed as an effective, safe, and easy method of detection in Australia [14], Argentina [15], Uruguay [16], or Peru [17]. This study provides a reference for improving control measures and assessment of the prevalence of echinococcosis in the endemic counties of China, a key step towards elimination. Since the most sensitive indicator of epidemic risk is dog infection rate, these kits are tools of primary importance. The results of this study were made public by the echinococcosis control programme office in order to provide guidance to all endemic provinces. With the aim of providing long-term and continuous support and guidance, it was suggested that national authorities should carry out a test every two years to objectively evaluate the control efficacy. The sandwich ELISA is fast, cheap, and easy to implement. However, the main problem remains the sensitivity and specificity of the current tests, which are not high enough. The sensitivity of reagents is a critical issue. In general, the fewer worms there are, the less likely samples will be positively detected. Therefore, we suggested to improve the sensitivity by collecting multiple samples from one dog or collecting multiple samples at one time, or using parallel detection with two different kits at the same time. Finally, we urge manufacturers to strengthen research on their products in order to improve and enhance their overall quality, in particular sensitivity and specificity, for effective *Echinococcus* diagnosis and control implementation in China.
